# Development of job-exposure matrices for trichloroethylene, tetrachloroethylene, and formaldehyde in Chinese workplaces

**DOI:** 10.1093/annweh/wxag076

**Published:** 2026-11-05

**Authors:** Jia Nie, Calvin B Ge, Melissa C Friesen, Lauren M Hurwitz, Wei Hu, Nathaniel Rothman, Qing Lan, Roel Vermeulen, Susan Peters

**Affiliations:** Institute for Risk Assessment Sciences, Utrecht University, Yalelaan 2, 3584 CM Utrecht, The Netherlands; Institute for Risk Assessment Sciences, Utrecht University, Yalelaan 2, 3584 CM Utrecht, The Netherlands; Occupational and Environmental Epidemiology Branch, Division of Cancer Epidemiology and Genetics, National Cancer Institute, NIH, DHHS, Bethesda, MD 20892, United States; Occupational and Environmental Epidemiology Branch, Division of Cancer Epidemiology and Genetics, National Cancer Institute, NIH, DHHS, Bethesda, MD 20892, United States; Occupational and Environmental Epidemiology Branch, Division of Cancer Epidemiology and Genetics, National Cancer Institute, NIH, DHHS, Bethesda, MD 20892, United States; Occupational and Environmental Epidemiology Branch, Division of Cancer Epidemiology and Genetics, National Cancer Institute, NIH, DHHS, Bethesda, MD 20892, United States; Occupational and Environmental Epidemiology Branch, Division of Cancer Epidemiology and Genetics, National Cancer Institute, NIH, DHHS, Bethesda, MD 20892, United States; Institute for Risk Assessment Sciences, Utrecht University, Yalelaan 2, 3584 CM Utrecht, The Netherlands; Institute for Risk Assessment Sciences, Utrecht University, Yalelaan 2, 3584 CM Utrecht, The Netherlands

**Keywords:** occupational exposure, trichloroethylene, tetrachloroethylene, formaldehyde, job-exposure matrices, China

## Abstract

**Background:**

Job-exposure matrices (JEMs) provide a systematic method to assign exposures based on job titles, thereby enabling efficient occupational exposure assessment in observational studies. However, few JEMs have been specifically developed for chemical occupational exposures in China.

**Objectives:**

We aimed to construct semi-quantitative JEMs for trichloroethylene (TCE), tetrachloroethylene (PCE), and formaldehyde in the Chinese context. These JEMs were developed to support occupational exposure assessment needs in the AsiaLymph study, and to highlight key methodological considerations in JEM construction.

**Methods:**

Exposure information was synthesized from the published literature, routine industrial hygiene inspection data retrospectively collected as part of the AsiaLymph study (2012 to 2017), and established international JEMs. The JEMs were structured across 3 dimensions: chemical agents (TCE, PCE, formaldehyde), job titles (ISCO-88, 3- and 4-digit), and calendar period (1980 to 1990, 1991 to 2000, 2001 to 2010, ≥2011). For each chemical-job-period cell, 2 epidemiologists independently assigned exposure probability and level using a harmonized semi-quantitative scale. Consensus estimates across chemicals and inter-rater agreement were evaluated using Spearman's rank correlation coefficients and Cohen's κ statistics, respectively.

**Results:**

Among 478 job titles, non-zero exposure probability was assigned to 116 (24%) for TCE, 109 (23%) for PCE, and 118 (25%) for formaldehyde. When applying a more stringent, high-specificity exposure definition (probability = 1), the number of job titles classified as exposed decreased to 31 (7%), 25 (5%), and 30 (6%), respectively. Consensus exposure probabilities showed the strongest correlation between TCE and PCE (ρ = 0.95). Inter-rater agreement for exposure probability was 0.90 for TCE, 0.78 for PCE, and 0.44 for formaldehyde.

**Discussion:**

Our semi-quantitative JEMs combine empirical data with expert judgment, offering a practical tool to assess occupational exposures in observational studies. While developed to address the specific needs of the Asialymph study, the JEMs are adaptable and can be validated and extended to other chemical agents in similar settings.

What's important about this paper This study presents the first semi-quantitative job-exposure matrices (JEMs) for trichloroethylene, tetrachloroethylene, and formaldehyde tailored to Chinese workplaces. By integrating diverse exposure data with expert judgment, the JEMs offer a practical approach for assessing occupational exposures when measurement data are limited and support epidemiological studies of cumulative exposures and long-term health outcomes. The JEMs can be further validated, refined, and extended to other chemicals and settings.

## Introduction

Accurate exposure assessment is critical in epidemiological research estimating exposure-disease relationships. In occupational settings, while individual-level exposure measurements are often regarded as the gold standard, they are resource-intensive, impractical for large observational studies, and unsuitable for retrospective exposure assessment. Job-exposure matrices (JEMs) were therefore introduced as a practical alternative, enabling standardized and efficient exposure assessment by systematically assigning exposure estimates based on job titles (Hoar 1983).

JEMs have been widely developed, applied, and harmonized in Western countries for a wide range of chemical agents (Peters et al. 2022; Selander et al. 2025). In contrast, few JEMs exist that account for China's historical industrial practices. The few existing examples are mainly limited to specific cohorts or particular regions, including JEMs for benzene and lead for Shanghai population-based cohorts and a silica dust JEM developed for Shanghai (Zhu et al. 2010; Friesen et al. 2012; Koh et al. 2014). This limited availability of JEMs constrains the systematic assessment of occupational exposures and their associated health effects in Chinese populations.

Trichloroethylene (TCE), tetrachloroethylene (PCE), and formaldehyde are widely used industrial solvents in China. TCE has been extensively used in electronics cleaning and metal degreasing, with measurements indicating consistently high exposure levels over several decades (Nie et al. 2025a). PCE has primarily been used as a cleaning agent in the dry-cleaning industry. Formaldehyde exposure has been documented in a range of occupations, including wood processing, chemical and water-treatment plants operating, painting, metal molding, plastics manufacturing, and scenarios using formaldehyde for disinfection and preservation (Nie et al. 2025b). Various health effects of these agents have been identified. For instance, evaluations by the International Agency for Research on Cancer (IARC) have classified TCE as a Group 1 human carcinogen with sufficient evidence for kidney cancer, PCE as a Group 2A probable human carcinogen linked to bladder cancer, and formaldehyde as a Group 1 human carcinogen associated with nasopharyngeal cancer and leukemia (Cogliano et al. 2004; Guha et al. 2012).

This study aimed to construct semi-quantitative JEMs for TCE, PCE, and formaldehyde tailored to Chinese occupational contexts while also providing a generalizable framework for future JEM development and application. The 3 exposures were selected to support exposure assessment for the AsiaLymph study, a large multicenter hospital-based case–control study of lymphoid and myeloid neoplasms in East Asian populations (Hurwitz et al. 2026).

## Methods

The development of the JEMs involved defining a structured framework, integrating exposure information, and assigning exposure probability and level.

### JEM structure

The JEMs were organized across 3 dimensions:

1. Chemical agents: TCE, PCE, and formaldehyde.
2. Job titles: Job titles (*n* = 478) coded at the 3- and 4-digit levels using the International Standard Classification of Occupations (ISCO-88) (International Labour Organization 2024).
3. Calendar periods: Exposure was estimated for four time periods (1980 to 1990, 1991 to 2000, 2001 to 2010, and ≥2011) to account for temporal changes in industrial practices and regulations. Exposure measurement data before 1980 was scarce and insufficient for reliable estimation, so 1980 was used as the starting point. The post-2011 period was collapsed into a single category to align with the intended application of the AsiaLymph study (2012 to 2017).

Two exposure parameters were predefined:

1. Exposure probability: The probability of workers in a given job title expected to be exposed, which were categorized as 0, 0.1, 0.25, 0.5, 0.75, and 1. These values were intended as practical probability scores rather than empirical percentiles or a symmetric scale. A value of 0.1 was included to capture jobs with possible but rare exposure, whereas a value of 0.9 was not included. Given that exposure probability was mostly right skewed, it was difficult to distinguish reliably from 1 and could imply a degree of precision not supported by the data.
2. Exposure level (mg/m^3^): The exposure level was coded from 0 to 4. A code of 0 indicated no or negligible exposure, and higher codes indicated higher exposure. The cut-offs were based on approximate quartiles of measurement data among exposed jobs and were rounded to practical values. The categories were 0, >0 to <30, ≥30 to <100, ≥100 to <200, and ≥200 mg/m^3^ for TCE; 0, >0 to <0.5, ≥0.5 to <50, ≥50 to <200, and ≥200 mg/m^3^ for PCE; and 0, >0 to <0.2, ≥0.2 to <0.5, ≥0.5 to <1.5, and ≥1.5 mg/m^3^ for formaldehyde.

### Data sources and assessment process

To estimate occupational exposure probability and level across job-chemical-period combinations, we synthesized and harmonized exposure information from four major sources:

1. Pooled concentrations by job titles based on literature and routine industrial hygiene inspection datasets for TCE (Nie et al. 2025a), PCE (Tables S1–2 and Supplementary Materials S1–5), and formaldehyde (Nie et al. 2025b) in Chinese workplaces.
2. International JEMs including the ALOHA+ JEM for chlorinated solvents for TCE and PCE (de Jong et al. 2014) and MatEmESp JEM for TCE, PCE, and formaldehyde (García et al. 2013).
3. A published list of job titles with potential occupational formaldehyde exposure (Fang et al. 2009).
4. Expert assessment (J.N., C.G., S.P., R.V.).

Information from sources 1 to 3 was first extracted and mapped to ISCO-88 job codes wherever possible. We regarded all identified job titles as potentially exposed. To ensure comprehensive coverage, we then expanded this initial set by retrieving all neighboring 4-digit job titles within the same upper-level 3-digit occupational groups. This process resulted in 190 job titles for TCE, 183 for PCE, and 304 for formaldehyde.

Exposure measurements were standardized to mg/m^3^ and grouped by chemical, job title, and calendar period. Measurement data was used mainly to guide exposure levels, while international JEMs and published list of job titles were used to support exposure probability assignment, especially for jobs with limited Chinese data. Two raters (J.N. and C.G.) independently estimated exposure probability for each unique chemical-job combination and exposure level for each unique chemical-job-period combination. Discrepancies were discussed and resolved with S.P. and R.V. to reach an adjusted consensus.

### Statistical analysis

To evaluate the similarity of exposure probability patterns across chemicals, pairwise Spearman's rank correlation coefficients (ρ) were computed using the consensus exposure probabilities for TCE, PCE, and formaldehyde across job titles.

Agreement between the estimates from 2 raters was evaluated separately for each chemical using Cohen's kappa statistic (κ) (Warrens 2012). Two forms of κ were calculated for exposure probability: (i) Unweighted Cohen's κ based on binary classification, where probabilities were dichotomized into exposed (≥0.5) and non-exposed (<0.5); (ii) Quadratically weighted Cohen's κ, where probabilities were treated as 6 ordered categories (0, 0.1, 0.25, 0.5, 0.75, and 1). Agreement in assessed exposure probabilities between the 2 raters and the adjusted consensus was visualized using heatmaps. For exposure levels, agreement was assessed for each calendar period and pooled across periods using quadratically weighted Cohen's κ. The 95% confidence intervals for pooled κ values were obtained by nonparametric bootstrap resampling.

## Results

The final JEMs covered 478 distinct ISCO-88 job codes with estimated exposure probability and level for TCE, PCE, and formaldehyde across four periods. Overall, 116 (24%), 109 (23%), and 118 (25%) job titles had an estimated exposure probability ≥ 0.1 for TCE, PCE, and formaldehyde, respectively (Table 1). When applying a more stringent, high-specificity exposure definition (probability = 1), the numbers of job titles classified as exposed reduced to 31 (7%), 25 (5%), and 30 (6%), respectively.

**Table 1:** Number of ISCO-88 job titles estimated as exposed to TCE, PCE, and formaldehyde in Chinese workplaces.

Estimated probability of exposure	0.1	0.25	0.5	0.75	1	Total^a^
TCE	43 (9.0%)	22 (4.6%)	10 (2.1%)	10 (2.1%)	31 (6.5%)	116/478 (24.3%)
PCE	35 (7.3%)	21 (4.4%)	21 (4.4%)	7 (1.5%)	25 (5.2%)	109/478 (22.8%)
Formaldehyde	36 (7.5%)	22 (4.6%)	22 (4.6%)	8 (1.7%)	30 (6.3%)	118/478 (24.7%)

^a^A total of 478 3-digit and 4-digit ISCO-88 job titles were assessed for each chemical, armed force were not included.

Table 2 shows the Spearman's rank correlation matrix for consensus probabilities of exposure to these 3 chemicals across job titles. The strongest correlation was observed between TCE and PCE (ρ = 0.95), followed by PCE and formaldehyde (ρ = 0.57) and TCE and formaldehyde (ρ = 0.56).

**Table 2:** Spearman's rank correlation matrix of the consensus probability of exposure to TCE, PCE, and formaldehyde across job titles.

	TCE	PCE	Formaldehyde
TCE	1.00	0.95	0.56
PCE		1.00	0.57
Formaldehyde			1.00

Agreement on exposure probabilities between 2 raters was generally moderate to high (Fig. S1**)**. Weighted κ values were 0.90 for TCE, 0.78 for PCE, and 0.44 for formaldehyde, and were consistently higher than the corresponding binary κ values (0.87, 0.67, and 0.35, respectively). Agreement on exposure levels across calendar periods was generally higher for TCE and PCE, and lower for formaldehyde (Table S3).

## Discussion

We developed JEMs using measurement data, published studies, international JEMs, and structured expert evaluation, yielding harmonized estimates of exposure probability and level. To our knowledge, these represent the first chlorinated solvent- and formaldehyde-specific JEMs tailored to the Chinese occupational context that provides harmonized national estimates for job-based exposure assessment.

When using a sensitive exposure definition (probability ≥0.1), 116 (24%), 109 (23%), and 118 (25%) job titles were classified as potentially exposed to TCE, PCE, and formaldehyde, respectively; applying a more specific definition (probability = 1) reduced these numbers to 31 (7%), 25 (5%), and 30 (6%), respectively, indicating that high-certainty exposure was confined to a limited set of occupations. Moreover, the strongest rank correlation of exposure probability observed between TCE and PCE were consistent with their historical co-use in metal-degreasing and dry-cleaning industries (Doherty 2000a, 2000b). The high-probability chlorinated solvent-exposed job titles identified in our JEMs showed qualitative overlap with exposed occupational groups in the NOCCA-JEM (Kauppinen et al. 2009). For example, electronics and telecommunications workers, electrical and electronic equipment assemblers, painters, printers, and laundry workers were identified in both JEMs. However, differences in assigned exposure probabilities, exposure levels, or temporal trends between the NOCCA-JEM and our JEMs may reflect both methodological differences, including occupational coding systems and calendar coverage, and differences in industrial settings between China and Nordic countries.

Agreements between the probabilities assigned by the 2 occupational epidemiologists were generally high for these 3 chemicals. The comparatively weaker agreement for formaldehyde was primarily driven by non-zero probabilities assigned by only one rater to occupations with diesel engine exhaust exposure. Formaldehyde can occur as a combustion by-product in diesel exhaust (Chin et al. 2012), but such exposures are generally low-level compared with exposures arising from specific applications. Accordingly, the final consensus assignment for formaldehyde in our JEM excluded diesel engine exhaust exposures, and future applications may consider complementary exposure assessments for diesel engine exhaust available from other sources (Ge et al. 2020).

The current work has several strengths. The development process of the Chinese JEMs was transparent, with harmonized criteria applied systematically, and achieved moderate to high inter-rater agreement. The JEMs provide a comprehensive framework covering nearly 500 job titles across four decades, integrating both empirical data and expert judgement. They therefore enable retrospective exposure assessment in epidemiological datasets with occupational histories and support analyses of exposure–response relationships for chronic diseases in Chinese populations. In addition, the comprehensive exposure framework can also support cross-sectional exposure assessment for more acute health outcomes. For epidemiological applications, we recommend applying more stringent exposure probability (≥0.5) as cutoff for exposure to improve exposure specificity (Kromhout and Vermeulen 2001). For continuous analyses, we recommend to use pre-specified semi-quantitative values based on category midpoints and 5/3 of the lower bound for the open-ended highest category.

Several limitations should be considered when applying these JEMs. Potential misclassification is possible because estimates depend on the completeness and representativeness of underlying data sources, including measurement data, literature reports, and expert judgment. For example, the relatively small number of PCE measurements limits the precision and stability of the assigned exposure level. In addition, external validation against independent measurement data was not possible in the current study. Second, between-worker variability is not captured; uniform exposure assignments could mask individual differences in tasks or work practices, as experts warning that “JEMs are no magic fix” for exposure assessment (Peters 2020). When JEMs are used in epidemiological analysis, such misclassification is typically non-differential with respect to disease status and is primarily Berkson-type error, whereby group-level exposure assignment reduces the precision of exposure–disease association estimates (Kauppinen 1994). Moreover, exposure levels are semi-quantitative, providing relative rather than absolute exposure levels. Future refinements could include calibration with independent measurement datasets and incorporating job-industry or job-task combinations. Nevertheless, JEMs remain a powerful tool for large-scale occupational exposure assessment, enabling exposure–response analysis in populations where individual-level exposure measurements are not feasible.

Overall, the developed JEMs provide a foundation for exposure assessment in Chinese workplaces and will support future epidemiological research within the AsiaLymph Study, as well as other studies in East Asia, while also offering a framework that can be further validated and refined as more data become available.

## Supplementary Material

wxag076_Supplementary_Data

## Data Availability

Data and job-exposure matrices developed in this study are available upon request from the corresponding author.
