# Supplementary material for: Development of job-exposure matrices for trichloroethylene, tetrachloroethylene, and formaldehyde in Chinese workplaces
Source: Ann Work Expo Health. 2026 Nov 5;70(8):wxag076. doi: 10.1093/annweh/wxag076 (PMC13738068; doi:10.1093/annweh/wxag076)
Supplement: Supplementary file 1 [file wxag076_supplementary_data.pdf]

# Supplementary Materials for “Development of Job-Exposure Matrices for Trichloroethylene, Tetrachloroethylene, and Formaldehyde in Chinese Workplaces”

Jia Nie[1], Calvin B. Ge[1], Melissa C. Friesen[2], Lauren M. Hurwitz[2], Wei Hu[2], Nathaniel Rothman[2], Qing Lan[2], Roel Vermeulen[1], and Susan Peters[1]*

[1] Institute for Risk Assessment Sciences, Utrecht University, Utrecht, the Netherlands

[2] Occupational and Environmental Epidemiology Branch, Division of Cancer Epidemiology and Genetics, National Cancer Institute, NIH, DHHS, Bethesda, MD, USA

*Author to whom correspondence should be addressed. Email: s.peters@uu.nl

***Supplementary figure 1. Agreement in the Assessed Probabilities Between Two raters and the Adjusted Consensus (CONS).***

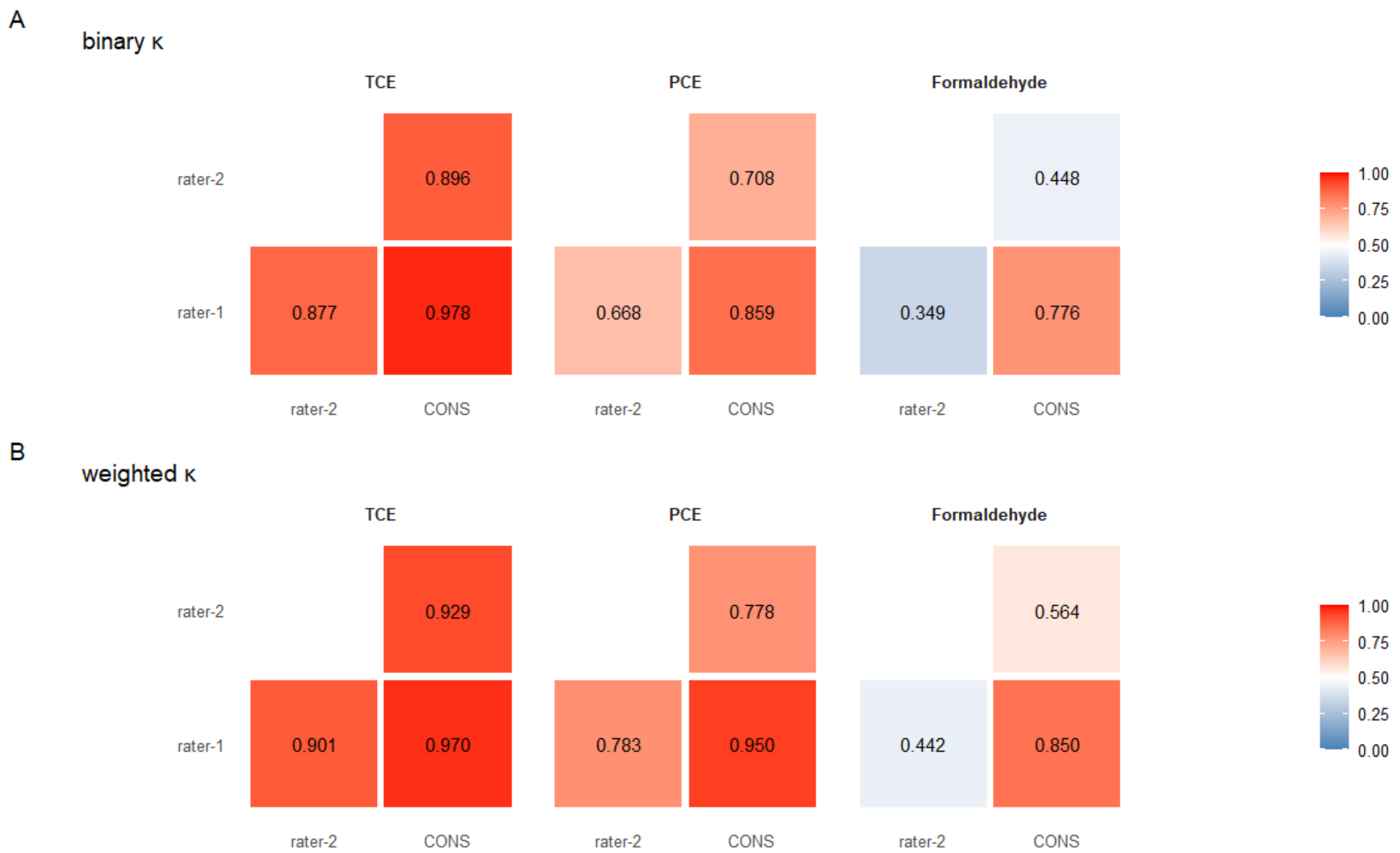


**Panel A:** Binary κ, calculated after dichotomizing probabilities at a ≥0.5 threshold (exposed vs not exposed).
**Panel B:** Weighted κ, calculated from six ordered probability categories (0, 0.1, 0.25, 0.5, 0.75, 1) using quadratic weights.
Each tile shows Cohen’s κ value; colors represent agreement strength from low (blue) to high (red).

***Supplementary table 1. Pooled mean tetrachloroethylene concentrations (mg/m$^3$) from the published literature grouped by 4-digit ISCO-88 job titles in China, 1983-2018.***

| Job title (ISCO-88) | Task-based* | No. measurements | Full-shift* | No. measurements |
|---|---|---|---|---|
| Varnishers and related painters (7142) | | | 7.03 (5.90-9.20) | 3 |
| Blacksmiths, tool-makers and related trades workers (7220) | | | 4.04 (0.26-10.8) | 9 |
| Metal wheel-grinders, polishers and tool sharpeners (7224) | 0.40 (0.40-0.40) | 12 | 0.40 (0.40-0.40) | 12 |
| Aircraft engine mechanics and fitters (7232) | 341 (332-351) | 2 | | |
| Compositors, typesetters and related workers (7341) | | | 1.95 (0.90-3.00) | 2 |
| Incinerator, water-treatment and related plant operators (8163) | 0.15 (0.15-0.15) | 9 | | |
| Metal finishing-, plating- and coating-machine operators (8223) | 224 (224-224) | 1 | | |
| Photographic-products machine operators (8224) | 64.2 (1.00-244) | 95 | | |
| Bleaching-, dyeing- and cleaning-machine operators (8264) | 242 (17.0-776) | 9 | | |
| Fur- and leather-preparing-machine operators (8265) | 1356 (678-2035) | 2 | | |
| Electrical-equipment assemblers (8282) | 33.9 (33.9-33.9) | 1 | | |
| Electronic-equipment assemblers (8283) | 411 (0-1017) | 8 | | |
| Hand-launderers and pressers (9133) | 120 (0-5679) | 3400 | 48.2 (0.69-230) | 1195 |
| Unknown | 0.44 (0.13-0.60) | 6 | 0.41 (0.03-0.60) | 6 |

* Weighted mean concentrations (minimum-maximum) were calculated for all measurements from both publications and representative measurements, and the minimum and maximum of the reported concentrations were shown in brackets. Methods used for the pooled mean tetrachloroethylene concentrations were listed in Supplementary Material 1-5.

***Supplementary table 2. Pooled mean tetrachloroethylene concentrations (mg/m$^3$) from the published literature grouped by 3-digit ISCO-88 job titles and different historical periods in China.***

| Job title (3-digit ISCO-88) | Year period | Task-based* | No. measurements | Full-shift* | No. measurements |
|---|---|---|---|---|---|
| All | 1980-1990 | 5 (0-2035) | 321 | | |
| | 1991-2000 | 144 (0-1135) | 739 | | |
| | 2001-2010 | 116 (0.13-5679) | 2404 | 50.2 (0.03-230) | 1145 |
| | 2011 onwards | 28.9 (0.15-539) | 81 | 1.51 (0.26-10.8) | 82 |
| Painters, building structure cleaners and related trades workers (714) | 2011 onwards | | | 7.03 (5.90-9.20) | 3 |
| Blacksmiths, tool-makers and related trades workers (722) | 2011 onwards | 0.40 (0.40-0.40) | 12 | 1.96 (0.26-10.8) | 21 |
| Machinery mechanics and fitters (723) | 2011 onwards | 341 (332-351) | 2 | | |
| Printing and related trades workers (734) | 2011 onwards | | | 1.95 (0.90-3.00) | 2 |
| Power-production and related plant operators (816) | 2011 onwards | 0.15 (0.15-0.15) | 9 | | |
| Chemical-products machine operators (822) | 1980-1990 | 65.9 (1.00-244) | 96 | | |
| Textile-, fur- and leather-products machine operators (826) | 1980-1990 | 453 (17.0-2035) | 9 | | |
| | 2011 onwards | 407 (275-539) | 2 | | |
| Assemblers (828) | 1980-1990 | 415 (33.9-10179) | 8 | | |
| | 1991-2000 | 0 | 1 | | |
| Domestic and related helpers, cleaners and launderers (913) | 1980-1990 | 111 (0.00-2035) | 208 | | |
| | 1991-2000 | 144 (0.00-1135) | 738 | | |
| | 2001-2010 | 116 (0.30-5679) | 2402 | 50.3 (5.21-230) | 1143 |
| | 2011 onwards | 16.1 (2.47-66.6) | 52 | 1.06 (0.69-2.62) | 52 |
| Unknwon | 2001-2010 | 0.13 (0.13-0.13) | 2 | 0.03 (0.03-0.03) | 2 |
| | 2011 onwards | 0.60 (0.60-0.60) | 4 | 0.60 (0.60-0.60) | 4 |

*Weighted mean concentrations (minimum-maximum) were calculated for all measurements from both publications and representative measurements, and the minimum and maximum of the reported concentrations were shown in brackets. Methods used for the pooled mean tetrachloroethylene concentrations were listed in Supplementary Material 1-5.

***Supplementary table 3. Agreement in Level of Exposure Assessment Between Epidemiologists (rater 1 and rater 2) and Consensus, by Chemical and Period***

| Chemicals | Periods | κ values between rater 1 and rater 2 | κ values between rater1 and CONS | κ values between rater 2 and CONS |
|---|---|---|---|---|
| TCE | 1980-1990 | 0.628 | 0.852 | 0.767 |
| | 1991-2000 | 0.690 | 0.935 | 0.768 |
| | 2001-2010 | 0.729 | 0.932 | 0.790 |
| | ≥2011 | 0.696 | 0.905 | 0.783 |
| | Pooled* | 0.676 (0.609-0.729) | 0.901 (0.865-0.932) | 0.774 (0.718-0.818) |
| PCE | 1980-1990 | 0.648 | 0.906 | 0.689 |
| | 1991-2000 | 0.623 | 0.905 | 0.692 |
| | 2001-2010 | 0.652 | 0.913 | 0.658 |
| | ≥2011 | 0.625 | 0.900 | 0.589 |
| | Pooled* | 0.637 (0.578-0.691) | 0.907 (0.878-0.938) | 0.662 (0.609-0.713) |
| Formaldehyde | 1980-1990 | 0.435 | 0.813 | 0.559 |
| | 1991-2000 | 0.456 | 0.832 | 0.514 |
| | 2001-2010 | 0.373 | 0.845 | 0.487 |
| | ≥2011 | 0.418 | 0.828 | 0.590 |
| | Pooled* | 0.428 (0.338-0.511) | 0.829 (0.780-0.874) | 0.535 (0.450-0.611) |

Notes: κ values represent quadratically weighted Cohen's kappa, measuring agreement in ordinal exposure level ratings (0 = none, 1 = low, 2 = medium, 3 = high, 4 = very high) between pairs (rater 1 and rater 2; rater 1 and CONS; rater 2-CONS) for each chemical and time period.

* Pooled values represent agreement across all periods combined, and 95% confidence intervals were obtained via nonparametric bootstrap resampling.

## ***Supplementary Material 1. Methods used for the pooled mean tetrachloroethylene concentrations listed in supplementary table 1 and 2.***

Comprehensive searches of electronic databases were conducted in both the English and Chinese languages. For English, PubMed, Embase, and Web of Science were searched. For Chinese, searches were conducted in the China National Knowledge Infrastructure database, the China Science and Technology Journal Database (VIP), and the WanFang database. Search methods were adjusted according to the different Chinese and English databases. The search included all the indexed papers and computerized literature databases, supplemented by manual screening reference lists from each relevant article.

Furthermore, representative measurements were extracted from a database containing task-based and full-shift personal and area measurements collected between 1983 and 2018 as part of the routine occupational hazard monitoring in Tianjin and Hong Kong. The PRISMA flowchart of data extraction is shown in **Supplementary Material 2**.

### *Search strategy*

Search Terms used for the literature review were: All fields= ((occupational AND exposure) OR (industrial AND exposure) OR (work AND exposure) OR (occupational AND air AND concentration) OR (occupational AND hazard) OR (occupational AND health) OR (industrial AND hygiene)) AND (tetrachloroethylene OR tetrachloroethene OR perchloroethylene OR perc OR PCE) AND (China OR Chinese OR Taiwan OR Hong Kong). The search was done on April 3rd, 2024. The search strings in Chinese used in different Chinese databases are listed in **Supplementary Material 3**.

### *Eligibility criteria*

Any publication reporting tetrachloroethylene (PCE) measurements conducted at workplaces in China was considered. We restricted our inclusion to publications in English or Chinese. We had no restriction on the time period. The most complete report was included when multiple publications were based on the same dataset. Publications or measurements related to acute poisonings, extremely high PCE concentrations that would be immediately dangerous for health, or duplicate publications in different journals were excluded.

### *Data extraction and synthesis*

The relevant information was extracted from publications or measurement datasets. For each identified set of summary statistics (any form of concentration level including single values, means with or without standard deviations, quantiles, ranges, etc.), we extracted related information including industry, job title and/or job task, sample year, location, area or personal sampling, number of measurements and other available information listed in **Supplementary Material 4**. All the extracted information was translated, coded, and tabulated. Short-term exposure level (STEL) and time-weighted average (TWA) concentration were defined as task-based and full-shift concentration, respectively; when none specified, we defined the type of concentration based on other information such as sampling duration. The job titles and industries were coded according to the International Standard Industrial Classification of All Economic Activities (ISIC4) (United Nations. 2008) and the International Standard Classification of Occupations (ISCO88) (International Labour Organization. 2010).

### *Analysis*

For each extracted set of summary statistics, the arithmetic mean (AM) was calculated for summarization, as it was the predominant form of reported concentration. If a single PCE concentration value was reported, it was considered as an individual measurement. If the authors only provided a range of values, the AM of the minimum and maximum reported values was used as the average. If only a maximum value was provided, the AM was calculated as the average of the maximum and zero. If only the minimum value was given, the observation was considered missing. When only the median and/or geometric mean (GM) were provided, it was used as the AM as only few publications reported these statistics. When “undetected” was reported, the value was set as 0.

We pooled the AM concentrations of each job title weighted by the number of measurements reported for the summary statistics. This was calculated by multiplying the number of sites measured by the number of samples taken per site. In circumstances where only one of these metrics was provided (either the number of sites measured or the number of samples taken per site), it was assumed that at least that many measurements were taken. In circumstances where none of the abovementioned sampling information was given, we assigned “at least 1” for a single concentration value and “at least 2” for a range of concentration or a mean value with standard deviation.

***Supplementary Material 2. PRISMA flowchart of the literature search and data screening process.***

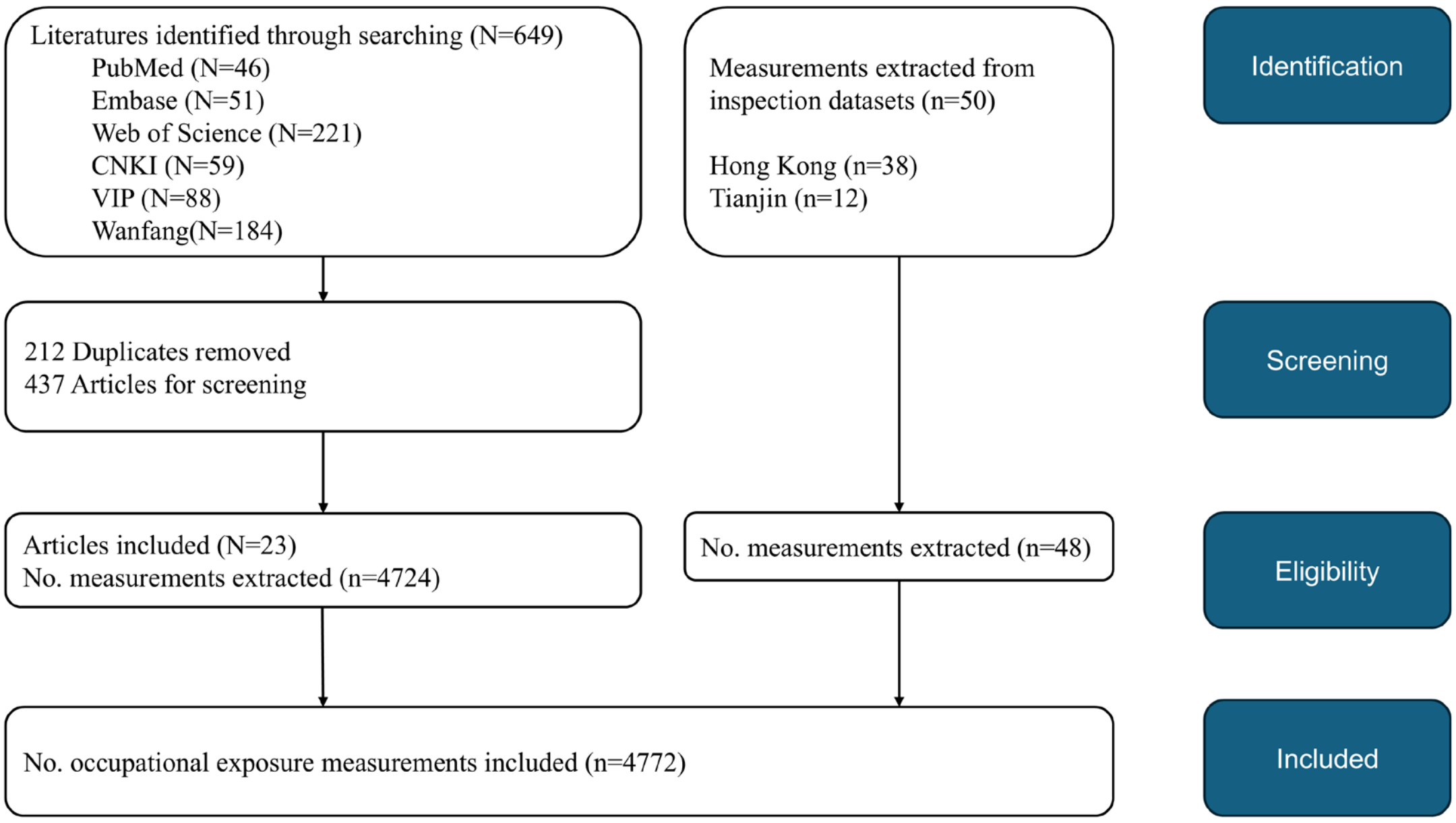

***Supplementary Material 3. Search terms used in different databases***

Search terms used in CNKI:
篇关摘=((职业 * 暴露) + (工业 * 暴露) + (工作 * 暴露) + (工作环境 * 空气浓度) + (职业卫生) + (职业健康) + (职业风险) + (职业危害)) * (四氯乙烯 + 全氯乙烯 + PCE + perc)

Search terms used in VIP
任意字段=((职业 * 暴露) + (工业 * 暴露) + (工作 * 暴露) + (工作环境 * 空气浓度) + (职业卫生) + (职业健康) + (职业风险) + (职业危害)) * (四氯乙烯 + 全氯乙烯 + PCE + perc)

Search terms used in Wanfang:
检索表达式（主题词扩展）： 全部=((职业 * 暴露) + (工业 * 暴露) + (工作 * 暴露) + (工作环境 * 空气浓度) + (职业卫生) + (职业健康) + (职业风险) + (职业危害)) and 全部=(四氯乙烯 + 全氯乙烯 + PCE + perc)

***Supplementary Material 4. information extracted from publications***

| Information extracted | Description |
|---|---|
| Summary statistics | For concentrations reported as TWA, STEL, or non-specified mean concentrations in literature, we further categorized them as full-shift, task-based, or other concentrations, respectively. AM, SD, median, ranges, GM, and GSD were extracted wherever possible. Concentrations in the unit of ppm were transferred to $mg/m^3$ (1 ppm = 6.78 $mg/m^3$) |
| Industry | Company name or the type of industry or products they made. |
| Job/task | Job titles. Job tasks were extracted if job titles were not reported and then coded by occupational epidemiology researchers. |
| Location | The province or city. |
| Sample reason | Type of study for scientific publications. Industrial hygiene surveys, occupational hazard assessments, environmental air quality inspections at workplace, or other types. |
| Sample year | Year of sampling. When the sample year was not provided, the year of publication was used. |
| Type of sample | Area or personal sampling. |
| Number of sites measured | The number of sites selected per industry or job. |
| Number of samples per site | The number of samples tested per industry or job. |
| Number of measurements | Equals to the number of sites * the number of samples taken per site. |
| Sampling and analytic method | Description of sampling and analytic methods. |
| Working conditions | Description of the availability of ventilation systems and other related protective procedures. |

Abbreviations: TWA=time weighted average, STEL=short term exposure level, AM=arithmetic mean, SD=standard deviation, GM=geometric mean, GSD=geometric standard deviation.

***Supplementary Material 5. Publications used for concentration extraction***


1. 万玲利, 黄琼, 陈固炤, 柯宗枝. 某服装干洗企业职业卫生现状调查[J]. 海峡预防医学杂志. 2017.
2. 任铁石, 任平平, 顾光霞. 干洗行业职业危害调查[J]. 河南预防医学杂志. 1999.
3. 冯慧民, 刘春华. 平顶山市服装干洗行业劳动卫生现状调查分析[J]. 平顶山师专学报. 2001.
4. 刘超, 毋尧, 王丽, 唐虹. 某纸加工企业职业病危害因素现状调查[J]. 职业与健康. 2012.
5. 卢伟, 朱彩菊, 孙广文, 倪蕴娥, 高金平. 上海部分中心城区洗衣行业职业卫生状况调查[J]. 劳动医学. 2000.
6. 周雪娥, 吕林萍, 李旭春, 董燕. 干洗行业职业卫生状况调查与分析[J]. 河北医药. 2008.
7. 崔京伟, 李长清, 杨伟瞻, 刘彦, 张志皋, 赵茜, 常元勋, 吕明刚, 王忱. 四氯乙烯作业工人血清中某些生化指标的变化[J]. 职业医学. 1998.
8. 张志皋. 洗衣工四氯乙烯中毒性周围神经病的劳动卫生调查报告[J]. 化工劳动保护：工业卫生与职业病分册. 1995.
9. 张志红, 何淑云, 叶琳, 高舫. 四氯乙烯对职业暴露人群外周血微核率的影响[J]. 中国饮食卫生与健康. 2004.
10. 张灶钦, 黎丽春, 闫雪华, 陈慧峰, 耿继武. 某飞机维修项目职业病危害和控制措施[J]. 中国卫生工程学. 2021.
11. 张瑞芳, 高淑敏, 任先云. 某羊剪绒制品生产企业职业病危害现状调查与评价[J]. 疾病监测与控制. 2015.
12. 徐希娴, 董家惠, 史志澄. 服装干洗作业者的健康调查[J]. 化工劳动保护(工业卫生与职业病分册). 1989.
13. 戴日英, 张志皋, 宋岩, 王秀荣, 李长青, 吴瑞华. 四氯乙烯作业工人劳动卫生调查[J]. 化工劳动保护：工业卫生与职业病分册. 1996.
14. 杜立华, 刘雅梅. 北京市部分洗衣房中四氯乙烯污染现状[J]. 职业与健康. 2005.
15. 梁戈清, 张雪军, 刘多见, 汤帏婷, 贾煜. 辽宁省某润滑油研发单位职业病危害因素调查[J]. 职业与健康. 2016.
16. 欧军荣, 江世强, 段平宁, 黎海红, 吕林, 许晓丽. 聚氯乙烯生产过程中的职业病危害及其控制[J]. 应用预防医学. 2006.
17. 汪晓媛, 耿月华, 刘雨. 聊城市干洗业职业危害调查[J]. 中国城乡企业卫生. 2004.
18. 焦贤芳, 岳峰勤, 苏保军, 关世忠. 郑州市干洗行业空气中四氯乙烯污染状况调查[J]. 职业与健康. 2005.
19. 王晓云, 张晶. 四氯乙烯健康危险度评价及其环境卫生标准研究[J]. 2003年环境与职业医学国际学术研讨会论文集. 2003.
20. 田东超, 翁少凡, 丘海丽, 谢子煌. 深圳市某污水处理厂职业病危害因素调查分析[J]. 职业与健康. 2016.
21. 蔡笃和, 周明岛, 牛永茂, 蒙绪发, 周红妹, 闵盛海. 一起急性三氯乙烯和四氯乙烯混合气体中毒事

故分析[J]. CHINA TROPICAL MEDICINE. 2002.
22. 许志恒, 卿文静, 林孟端, 林海端. 某机械五金有限公司职业病危害控制效果评价[J]. 职业与健康. 2016.
23. 钟桂普. 服装干洗业接触四氯乙烯的现况调查[J]. CHINESE JOURNAL OF INDUSTRIAL HYGIENE AND OCCUPATIONAL DISEASES. 2001.
